# The Synergic Effect of a Nutraceutical Supplementation Associated to a Mediterranean Hypocaloric Diet in a Population of Overweight/Obese Adults with NAFLD

**DOI:** 10.3390/nu14224750

**Published:** 2022-11-10

**Authors:** Martina Chiurazzi, Nunzia Cacciapuoti, Mariastella Di Lauro, Gilda Nasti, Margherita Ceparano, Elisabetta Salomone, Bruna Guida, Maria Serena Lonardo

**Affiliations:** Department of Clinical Medicine and Surgery, Physiology Nutrition Unit, Federico II University of Naples, 80131 Naples, Italy

**Keywords:** hepatic steatosis, nutraceutical supplementation, obesity

## Abstract

Overweight/obesity is often associated with a non-alcoholic fatty liver disease (NAFLD). The study aim was to investigate the effects of a nutraceutical supplementation associated to a Mediterranean-hypocaloric-diet (MHD) on ultrasound-liver-steatosis (ULS) grade improvement in overweight/obese patients with NAFLD. A total of 68 subjects (BMI ≥ 25 kg/m^2^) with NAFLD were recruited, randomized into 2 groups and treated for 3 months: the Nutraceutical group was treated with MHD plus nutraceutical supplementation (Vitamin E, L-glutathione, silymarin and hepato-active compounds); the Control-group only with a MHD. Anthropometric measurements, body composition, biochemical parameters and Hepatic steatosis index (HSI) were evaluated at baseline and after 3 months; patients with HSI >36 underwent a liver ultrasound to determine liver steatosis grade (3 severe, 2 moderate, 1 mild). In all patients, a significant improvement in nutritional and biochemical parameters was observed after treatment. After treatment, the nutraceutical group showed a significant improvement in hepatic steatosis, either according to ULS-grade (11.1% and 5.6% of patients with mild and moderate liver steatosis, respectively, showed a complete NAFLD regression; 33.3% and 22.2% of patients with moderate and severe liver steatosis, respectively showed a regression to mild liver steatosis), or according to HSI (49.3 ± 10.1 vs. 43.3 ± 9.0, *p* = 0.01), suggesting that a healthy diet is still the best choice, although the use of specific supplements can enhance the efficacy of dietary intervention in overweight/obese patients with NAFLD.

## 1. Introduction

Obesity, characterized by a pathological accumulation of body fat, represents one of the greatest public health problems worldwide and is often associated with an increased risk of non-alcoholic fatty liver disease (NAFLD) [1]. NAFLD is defined when there is histologic evidence of hepatic steatosis alone, whereas non-alcoholic steatohepatitis (NASH) is defined when steatosis, lobular inflammation, and hepatocyte swelling—with or without perisinusoidal fibrosis—are observed. Furthermore, the overall mortality in NAFLD is significantly increased because of both cardiovascular and liver-related complications, that progress in end-stage liver disease, cirrhosis or hepatocellular carcinoma [2,3].

NAFLD is generally identified as the hepatic manifestation of the metabolic syn-drome (MetS). This condition, indeed, is often associated with type 2 diabetes mellitus (DM2), as well as dyslipidaemia (high plasma TG level and/or low plasma concentrations of HDL cholesterol) and hypertension [4,5,6].

The physiopathology of NAFLD is complex, multifactorial and not yet fully known; dietary factors as well as insulin resistance, genetic factors and alterations of the intestinal microbiota seem to be involved in the onset of NAFDL [7,8,9].

To date, the diagnosis of NAFLD is based on the exclusion of other causes of fatty liver disease, such as current or past consumption of significant amounts of alcohol (the European Association for the Study of the Liver, defines 30 g per day for men and 20 g per day for women as significant hepatotoxic alcohol amounts), and this is supported by laboratory tests and imaging techniques [10,11].

Elevated serum levels of transaminases such as Aspartate-aminotransferase (AST) and Alanin-aminotransferase (ALT) represent the most common biochemical parameters abnormality in NAFLD [12,13]. In addition to laboratory markers, physical measurements are used to diagnose the presence and severity of NAFLD. Ultrasonography, due to its common availability and low costs, still represents the first-line diagnostic tool for liver steatosis, although this technique is not able to detect minor steatosis or distinguish NASH from NAFLD; cirrhosis can only be diagnosed in advanced cases [4]. Liver biopsy represents the gold standard for assessing the severity of NAFLD, but it has well known limitations including invasiveness, rare but potentially life-threatening complications and cost [14,15].

The therapeutical goals in NAFLD patients aim for a slowing down or stopping of the disease progression and improving steatosis, in order to prevent inflammation and fibrosis [4].

To date, first-line therapy for NAFLD is based on lifestyle changes to induce weight loss, through caloric restriction and physical activity [16,17].

In 2017, Abenavoli L. et al. showed that the Mediterranean diet alone can improve anthropometric parameters and lipid profile as well as help to slow down hepatic fat accumulation and liver stiffness. Furthermore, the association of the Mediterranean diet with antioxidant supplementations can contribute to ameliorate the insulin sensitivity, supporting a possible role of antioxidant supplementation as a synergic therapy in patients with NAFLD [18]. Currently, there are no specific drugs approved for NAFLD/NASH therapy; moreover, drug treatments specifically able to improve liver disease are used only in advanced NASH patients with fibrosis. In recent years, increasing attention has been placed on the beneficial effects exerted by several nutraceuticals in the treatment of many diseases; different types of substances were suggested for the treatment of NAFLD/NASH [19].

Many studies, indeed, have reported the antioxidant, anti-inflammatory and hepatoprotective properties of some substances naturally present in foods and plants such as Vitamin E, L-Glutathione and Silymarin, suggesting their feasible role in aiding with many pathophysiological conditions [20]. In particular, some studies showed the effects of silymarin supplementation on the reduction of liver enzymes, serum biomarkers and ultrasonographic results in NAFLD patients, as well as the effects of vitamin E and glutathione supplementations on the improvement of liver abnormalities. Furthermore, some studies showed that the combination of silymarin with other antioxidant substances such as Vitamin C, Vitamin E, Coenzyme Q10, L-Glutathione and Selenomethionine could be able to improve silymarin efficacy [21]. In 2015, Aller R. et al. demonstrated that supplementation with silymarin plus vitamin E in a hypocaloric diet could improve hepatic function, a change also observed in patients without a 5% weight loss [22]. Therefore, in accordance with previous studies that show the hepatoprotective effects of substances such as Vitamin E, L-Glutathione and Silymarin in NAFLD, the aim of the present study was to investigate the synergic effects of a nutraceutical supplementation characterized by the association of Vitamin E, L-Glutathione and Silymarin combined with some hepato-active compounds such as Vitamin B12, L-methionine, L-Cysteine and Soy Phospholipidis associated also with a Mediterranean hypocaloric diet (MHD) on improvement of liver steatosis grade in patients with NAFLD [23,24,25,26,27,28,29,30,31].

## 2. Materials and Methods

### 2.1. Study Design

Double-blind randomized controlled clinical trial. The study protocol was approved by the Ethical Committee of the Federico II University Medical School of Naples (Project identification code 125/17) and all patients gave written informed consent.

Sixty-eight overweight/obese subjects with Body Mass Index (BMI) ≥25 kg/m^2^ and NAFLD attending the Outpatients Clinic of the Departmental program “Diet therapy in transplantation and chronic renal failure”, School of Medicine, “Federico II” University of Naples, were recruited.

After baseline evaluations, subjects were randomized into 2 groups and treated for 3 months: The first group (36 patients) was treated with a Mediterranean hypocaloric diet (MHD) plus nutraceutical supplementation (Vitamin E, L-glutathione, silymarin and hepato-active compounds) (Nutraceutical group); the second group (32 patients) was treated only with MHD (Control group).

The subjects belonging to the Nutraceutical group were treated for 3 months with 550 mg/die of nutraceutical supplementation characterized by Vitamin E, L-glutathione and silymarin plus hepato-active compounds (Vitamin B12, L-Methionine, L-Cysteine and Soy Phospholipidis) (Table 1).

The consumption of this nutraceutical supplementation is not associated with health risks. The supplement administered is registered in the “Register of supplements” of the Ministry of Health (code 86075).

Not taking pills for two or more days represented a criterion of exclusion from the study.

Patients were excluded when affected by diseases, such as intestinal malabsorption syndrome, chronic renal disease, cancer, thyroid diseases and other chronic liver diseases such as liver cirrhosis, chronic viral hepatitis and alcoholic hepatitis. Furthermore, patients with daily alcohol consumption were excluded from the study to avoid negative alcohol involvement on hepatic function and parameters. In particular, all patients with daily alcohol consumption (of more than 20 g and 30 g of alcohol for women and men, respectively, according to the European Association for the Study of the Liver) or who binge-drink during the weekends were excluded from the study. To test the compliance, food frequency questionnaires (FFQs) were administered to each patient in order to collect information on alcoholic beverage intake.

### 2.2. Study Protocol

**Figure 1:**
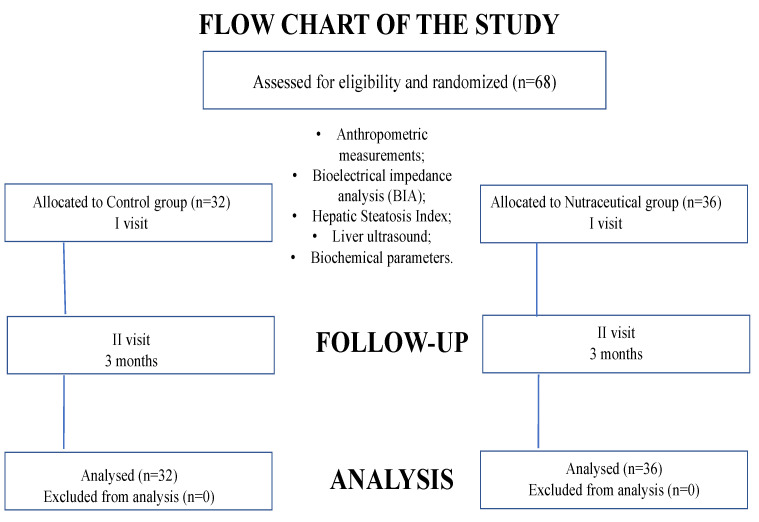
Flow-chart illustrating study population.

The subjects were evaluated at the moment of recruitment (time T0) and after 3 months (time T1) of treatment, using standardized protocols (Figure 1).

The nutritional status was assessed by anthropometric measurements: weight (Seca GmbH & Co KG, Hamburg, Germany), height (wall-mounted stadiometer to the nearest 0.1 cm), body mass index (BMI), waist circumference (WC) and hip circumference [32]. To evaluate the body composition, bioelectrical impedance analysis (BIA) was undertaken by tetrapolar BI (RJL 101; Akern SRL, Florence, Italy). BIA was performed with a single-frequency measurement (50 kHz) [33]. Steatosis index such as Hepatic Steatosis Index [HSI = 8 × (ALT/AST) + BMI + (2, if diabetes mellitus) + (2, if female), (with values < 30 ruling out and values > 36 ruling in steatosis)] was evaluated in both groups to predict the presence of hepatic steatosis [34,35]. The presence of diabetes mellitus was defined as a fasting glucose ≥ 126 mg/dL or treatment with anti-diabetic medication. Then, all patients with HSI > 36 were recruited and underwent liver ultrasound to determine the liver steatosis grade (3 severe, 2 moderate, 1 mild).

Blood parameters, such as blood glucose (Gly), total cholesterol (COL-tot), HDL cholesterol (Col- HDL), LDL cholesterol (Col-LDL), triglycerides (Try) and transaminases (AST and ALT) were measured and monitored during the treatment.

### 2.3. Dietary Treatment and Compliance

A personalized diet was recommended for each patient across both groups, according to the LARN (Livelli di Assunzione Raccomandata di Nutrienti) guidelines [36]. A Mediterranean hypocaloric diet (with a calorie restriction of 40% of the total energy needs), with 55–60% of the total caloric intake in carbohydrates (refined carbohydrates < 15%), 10–15% in proteins and 20–25% in lipids (<7% from saturated fat), has been recommended for all overweight/obese patients [37].

Compliance with the dietary intervention and nutraceutical supplementation were assessed, respectively, by monitoring the dietary intake through a Food Frequency Questionnaires and by a questionnaire asking each volunteer to record the time of consumption of the supplement, at baseline and at the end of the study.

### 2.4. Statistical Analysis

All data were expressed as mean ± standard deviation of the mean (DS). Data were analysed with the SPSS program (Statistical Package for Social Science, SPSS Chicago, IL, USA). Paired samples *t*-test, independent samples *t*-test and chi-squared test were performed. The statistical significance was set at *p* < 0.05.

## 3. Results

All patients were accurately evaluated at baseline and were reconsidered after 3 months. No significant differences in all parameters were observed at the baseline among two groups with the exception of HDL-Cholesterol (*p* = 0.000 Control group vs. Nutraceutical group) as well as LDL-Cholesterol (*p* = 0.003 Control group vs. Nutraceutical group), LDL/HDL ratio (*p* = 0.01 Control group vs. Nutraceutical group) and TG/HDL ratio (*p* = 0.03 Control group vs. Nutraceutical group) (Table 2). On the basis of a dietary questionnaire, in the whole cohort, all patients had high adherence to the Mediterranean Diet (data not shown). At the end of the observations, the patients of both groups showed a significant improvement in nutritional status as well as in body composition, Ultrasound liver steatosis (ULS) grade, Hepatic steatosis index and biochemical parameters compared to their baseline (Table 3).

### 3.1. Nutraceutical Group (N Group)

Nutraceutical supplementation improved anthropometric parameters of patients belonging to N group, inducing a significant reduction in Body Weight (92.8 ± 19.6 vs. 100.3 ± 19.2 Kg, *p* = 0.000 T1 vs. T0) as well as in BMI (34.0 ± 8.4 vs. 36.7 ± 8.6 Kg/m^2^, *p* = 0.000 T1 vs. T0), waist circumference (103.7 ± 14.9 vs. 110.0 ± 15.4 cm, *p* = 0.000 T1 vs. T0) and hip circumference (114.0 ± 12.1 vs. 117.8 ± 13.9 cm, *p* = 0.002 T1 vs. T0) compared to baseline after 3 months of treatment. Finally, bioimpedance analysis showed a significant reduction in FM % (33.7 ± 13.6% vs. 36.9 ± 12.5%; *p* = 0.007 T1 vs. T0) in N group after 3 months of nutraceutical supplementation. In contrast, a significant increase in FFM % (66.0 ± 13.6% vs. 63.1 ± 12.5% *p* = 0.007 T1 vs. T0) and TBW % (49.3 ± 9.2% vs. 46.9 ± 8.7% *p* = 0.007 T1 vs. T0) compared to baseline was detected.

It is interesting to observe the improvement in biochemical parameters; in particular, significant reductions in Gly (93.0 ± 15.4 vs. 105.6 ± 25.9 mg/dL, *p* = 0.006 T1 vs. T0), Col-Tot (161.4 ± 31.4 vs. 207.9 ± 49.7 mg/dL, *p* = 0.000 T1 vs. T0), Col-LDL (143.0 ± 37.1 vs. 204.3 ± 56.9 mg/dL, *p* = 0.000 T1 vs. T0), Triglycerides (122.9 ± 43.4 vs. 159.4 ± 65.9 mg/dL, *p* = 0.004 T1 vs. T0), LDL/HDL-c ratio (3.6 ± 1.6 vs. 5.6 ± 3.1, *p* = 0.001 T1 vs. T0) and TG/HDL-c ratio (3.1 ± 1.6 vs. 4.3 ± 2.4, *p* = 0.001 T1 vs. T0) were observed compared to baseline. Conversely, no significant difference was detected in Col-HDL after 3 months of treatment. Furthermore, a reduction of both transaminases was detected compared baseline (AST: 25.0 ± 9.4 vs. 38.5 ± 15.6 UI/L, *p* = 0.000 T1 vs. T0; ALT: 29.4 ± 9.7 vs. 53.8 ± 28.8 UI/L, *p* = 0.004 T1 vs. T0).

**Figure 2:**
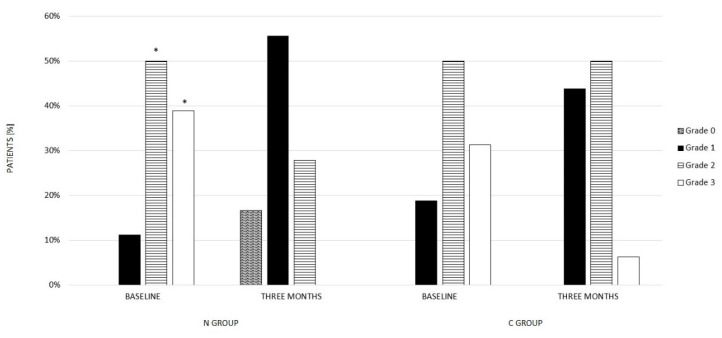
Ultrasound liver steatosis grade at baseline and after 3 months of treatment in N group and C group. Ultrasound liver steatosis grade: 0 = absent, 1 = mild, 2 = moderate, 3 = severe; * *p* < 0.05 vs. Three Months.

After 3 months of treatment, a significant difference was observed in HSI (43.3 ± 9.0 vs. 49.3 ± 10.1, *p* = 0.01 T1 vs. T0) compared to baseline. Furthermore, a significant improvement in the ULS grade was detected compared to baseline (Figure 2). At the end of the treatment, indeed, no patients showed severe-grade steatosis compared to the baseline (0% vs. 38.9%, T1 vs. T0), while six patients showed an absence of Hepatic steatosis (16.6% vs. 0%, T1 vs. T0). In particular, in 11.1% of patients who presented mild-grade steatosis at baseline, after 3 months of treatment with MHD and nutraceutical supplementation, there was a complete NAFLD regression, as well as in 5.6% of patients who showed moderate-grade liver steatosis at baseline. In 33.3% and 22.2% of patients who presented moderate- and severe-grade liver steatosis, respectively, a regression to mild-grade liver steatosis was observed after 3 months of treatment. Conversely, 11.1% of patients who presented moderate-grade liver steatosis at baseline did not show a difference after 3 months of treatment, while a regression to moderate-grade liver steatosis was detected compared to baseline in 16.7% of patients who showed severe-grade liver steatosis at baseline.

### 3.2. Control Group (C Group)

After 3 months of diet, patients belonging to C group showed a reduction in Body Weight (84.7 ± 12.2 vs. 92.1 ± 17.8 Kg, *p* = 0.000 T1 vs. T0) as well as in BMI (31.8 ± 5.7 vs. 33.9 ± 6.6 kg/m^2^, *p* = 0.009 T1 vs. T0), waist circumference (97.5 ± 11.0 vs. 104.1 ± 11.3 cm, *p* = 0.000 T1 vs. T0) and hip circumference (110.3 ± 11.8 vs. 113.1 ± 13.7 cm, *p* = 0.000 T1 vs. T0). Finally, bioimpedance analysis showed a significant reduction in FM % (32.6 ± 10.7% vs. 35.2 ± 11.4%; *p* = 0.009 T1 vs. T0) in C group after 3 months of MHD and an increase in FFM % (67.3 ± 10.8% vs. 62.7 ± 11.9%, *p* = 0.04 T1 vs. T0) and TBW % (50.5 ± 7.3% vs. 48.5 ± 7.8%, *p* = 0.006 T1 vs. T0).

After 3 months of MHD, patients showed a significant reduction in Gly (92.8 ± 13.8 vs. 101.1 ± 22.5 mg/dL, *p* = 0.02 T1 vs. T0) as well as in Triglycerides(105.5 ± 35.4 vs. 133.8 ± 53.0 mg/dL, *p* = 0.006 T1 vs. T0), Col-Tot (187.7 ± 36.0 vs. 213.1 ± 42.3 mg/dL, *p* = 0.02 T1 vs. T0), Col-LDL (150.2 ± 31.7 vs. 180.5 ± 41.2 mg/dL, *p* = 0.02 T1 vs. T0), TG/HDL-c ratio (1.8 ± 0.6 vs. 2.7 ± 1.8, *p* = 0.008 T1 vs. T0) and LDL/HDL-c ratio (2.6 ± 0.6 vs. 3.4 ± 1.4, *p* = 0.001 T1 vs. T0). No different variations were observed in Col-HDL compared to baseline. Furthermore, a reduction of both transaminases was detected after 3 months of MHD (AST: 24.6 ± 17.4 vs. 36.9 ± 13.0 UI/L, *p* = 0.002 T1 vs. T0; alt: 38.3 ± 10.6 vs. 54.0 ± 21.1 UI/L, *p* = 0.006 T1 vs. T0).

Finally, no significant differences were observed in HSI compared to baseline. On the other hand, an improvement in the ULS grade was observed compared to baseline (Figure 2); at the end of 3 months of MHD, indeed, only two patients still showed severe-grade liver steatosis compared to baseline (6% vs. 31.3%, T1 vs. T0). Differently from N group, no patients showed an absence of liver steatosis grade compared to baseline. In particular, in 3.2% of patients who presented severe-grade liver steatosis at baseline, 3 months of MHD induced a regression to moderate-grade liver steatosis. In 2.4% and 0.8% of patients, respectively, who presented moderate- and severe-grade liver steatosis, a regression to mild-grade liver steatosis was observed after 3 months of treatment. Conversely, 2.4% and 3.2% of patients who presented mild- and moderate-grade liver steatosis at baseline did not show a difference after 3 months of treatment. Moreover, o,8% of patients who showed moderate-grade liver steatosis at baseline showed a worsening to severe-grade liver steatosis after 3 months of MHD.

## 4. Discussion

The results of the present study indicate that overweight/obese subjects with different degrees of fatty liver disease, attending at Outpatients Clinic of the Departmental program “Diet therapy in transplantation and chronic renal failure”, School of Medicine, “Federico II” University of Naples, treated for 3 months with a Mediterranean hypocaloric diet (Control group) or with Mediterranean hypocaloric diet plus a supplement of 550 mg/day of Vitamin E, L-L-glutathione, silymarin and hepato-active compounds (Nutraceutical group), showed significant improvements in nutritional status, body composition, biochemical parameters and Liver steatosis (LS) grade compared to baseline. Furthermore, Nutraceutical group showed a significantly higher improvement in hepatic steatosis according to LS grade and HSI than the Control group.

In addition, our data demonstrated that after 3 months of treatment, patients belonging to the Nutraceutical group showed a significant reduction in Total Cholesterol and LDL Cholesterol compared to their baseline and to patients belonging to the Control group (*p* = 0.002 and *p* = 0.001, respectively).

Our data, therefore, show that the association between diet and a nutraceutical supplementation is able to induce greater effects, in particular on LS grade, than with dietary intervention alone. It is conceivable that these results can be related to the antioxidant, anti-inflammatory and hepatoprotective effects of vitamin E, L-L-glutathione and silymarin associated to hepato-active compounds. The results of the present study, support and are in agreement with previous studies demonstrating that natural substances such as Vitamin E, L-Glutathione and Silymarin are able to exert therapeutic effects in NAFDL.

Vitamin E is a potent antioxidant that appears to be able to reduce oxidative stress in NAFLD. Vitamin E is considered an important fat-soluble element, comprising a family of organic compounds including two isoforms, tocotrienol and tocopherol, that plays a fundamental role in the defence system within the cell. Several studies have shown that vitamin E is able to perform a powerful protective action against the complications of various diseases due to its antioxidant role, attributed to the hydroxyl group from the aromatic ring of tocochromanols, which is able to donate hydrogen to neutralize free radicals or reactive oxygen species (ROS) [24,38,39,40]. Furthermore, vitamin E is also involved in other activities, such as the regulation of inflammatory response, gene expression, membrane-bound enzymes, modulation of cellular signaling and cell proliferation [39]. Previous scientific data suggest that adjuvant vitamin E therapy provides significant biochemical (ALT and AST levels) and histological abnormalities (hepatic steatosis and lobular inflammation) improvements in adult patients with NAFLD, but further studies are needed to evaluate the long-term safety and efficacy of proposed treatments [38,40]. Indeed, to date, clinical studies that have tested the efficacy of vitamin E in both adults and children with NAFDL have reported contradictory results. In paediatric patients, vitamin E use did not show the same significant effects on liver function observed in adults. In contrast, the use of vitamin E in combination with other nutraceuticals as a treatment in NAFLD in children, showed promising results [26,38,41,42]. In 2021, Mosca et al. analyzed the plasma levels of markers of systemic inflammation in 70 children with NAFLD treated with vitamin E (10 mg) and hydroxytyrosol (7.5 mg) for 4 months, and showed that the combination of vitamin E and hydroxytyrosol induced an improvement in steatosis and hypertriglyceridemia [43]. In 2022, D’Espessailes A. et al., showed that Tocopherols (TF) supplementation in HFD-fed mice was able to modulate retinol metabolism, involved in the development of non-alcoholic fatty liver disease (NAFLD) through the reduction of the presence of lipid vesicles and total lipid content and the downregulation of the expression of hepatic retinaldehyde dehydrogenases such as RALDH1, RALDH3, SREBP-1c, and CD36 [44]. In 2022, Panera N. et al., through in vitro and in vivo approaches, demonstrated the potential therapeutic effect of Vit E alone or in combination with HXT to protect against NAFLD-related hepatic fibrosis, suggesting that supplementation with Vit E in combination with HXT can represent a potential therapeutic approach to improve NAFLD-related liver fibrosis, inducing a reduction of the disease progression risk [45].

On the other hand, many studies have revealed that glutathione introduced as a dietary supplement is able to exert numerous systemic effects such as the improvement of liver abnormalities and the improvement of diabetic complications [25,26]. In 2016, Irie et al., in a pilot study demonstrated that antioxidant therapy with three hundred milligrams per day of glutathione was able to reduce the pathological oxidative stress in the liver in NASH, preventing the progression from NAFLD to NASH [46]. Similarly, in 2017, Honda et al., in a pilot study on thirty-four NAFLD patients diagnosed using ultrasonography, showed the potential therapeutic effects of oral administration of glutathione in practical dose in being able to support hepatic metabolism and improve NAFLD [26].

Silymarin is extracted from the seeds of Silybum marianum L. Gaertn (also known as milk thistle); it is a mixture containing many flavonoids such as silybin, silidianin, silicristin and other active ingredients and exerts antioxidant, anti-inflammatory and hepatoprotective activity [27]. The most important hepatoprotective mechanism of silymarin is its activity as a free radical scavenger. In 2017, Marin V. et al. studied the beneficial effects of silymarin in a mouse model of non-alcoholic steatohepatitis (NASH). Mice supplemented with silymarin showed a substantial improvement in metabolic parameters (glycemia as well as lipid profile), but also in visceral fat, ALT, hepatic inflammation, oxidative stress and liver fibrosis [47]. In 2018, Hebat et al. showed the advantageous effects of silymarin in a rat model of NAFDL induced by a high-fat diet. Their data demonstrated that silymarin was able to reduce the biochemical and histopathological changes induced by high-fat diets in a rat model of NAFLD, inducing a significant reduction in serum cholesterol, triglycerides, AST and ALT in the treated group compared to the non-treated group [48].

In 2020, Kolota et al. demonstrated the influence of 3 months of therapy including dietary and physical activity recommendations combined with a daily milk thistle supply on the biochemical parameters of 20 NAFLD patients. Their results demonstrated that daily milk thistle supplementation in NAFDL patients was able to induce improvements in blood triglyceride levels and GGT activity even if other recommendations such as dietary and physical activity were not followed [27]. In 2021, Mengesha et al. investigated the hepatoprotective effect of silymarin on fructose-induced non-alcoholic fatty liver disease in rats. Their results showed that silymarin-treated groups presented an amelioration in oxidative stress, dyslipidaemia, and steatosis [49]. In 2020, Curcio A. et al., studied the efficacy and tolerability profile of the association of silymarin, vitamin C, vitamin E, coenzyme Q10 and selenomethionine on markers of liver function, and ultrasound results in 81 patients with mild to severe NAFLD. After 90 days of treatment, the treated group showed a significant reduction in ALT as well as AST, ALP, GGT, lipid parameters and an improvement of ultrasonographic results compared to the control group [21]. Furthermore, it has been shown that silymarin is able to increase the production of glutathione in the liver through an increase in the availability of its substrate such as cysteine; this activity can contribute to the enhancement of silymarin antioxidant capacity in liver tissues [50].

To date, no data have been reported on the protective effects of the combination of Vitamin E, L-Glutathione, silymarin and hepato-active compounds on NAFDL patients; based on this, the novelty of this study is represented by the results obtained from the synergy of these compounds on liver function.

In conclusion, a healthy diet remains the best choice in all conditions of metabolic alterations related to obesity. However, it is possible to use specific supplements to facilitate and enhance the body’s metabolic response to dietary treatment.

## Figures and Tables

**Table 1:** Chemical composition of nutraceutical capsules.

	Nutritional Information^®^
**Ingredients**	**Amount per Serving**
Tocopherol (Vit. E)	10 mg
Cyanocobalamin (Vit. B12)	1 mg
L-Methionine	100 mg
L-Cysteine	100 mg
L-Glutathione	50 mg
Soy Phospholipidis	40 mg
Silybum marianum E.S. tit. min.70% in silimarina	285.72 mg

**Table 2:** Baseline clinical characteristics of participants. Data are reported as mean ± DS. * *p* < 0.05 vs. Nutraceutical group.

	**Nutritional Assessment and Body Composition**	
	**Nutraceutical Group** **(n = 36)**	**Control Group** **(n = 32)**
**Male, n° (%)**	16 (44.4)	16 (50)
**Age, years**	56.1 ± 9.9	59.8 ± 10.9
**Weight, kg**	100.3 ± 19.2	92.1 ± 17.8
**BMI, kg/m^2^**	36.0 ± 8.6	33.9 ± 6.6
**Waist circumference, cm**	110.0 ± 15.4	104.1 ± 11.3
**Hip circumference, cm**	117.8 ± 13.9	113.1 ± 13.7
**TBW %**	46.9 ± 8.7	48.5 ± 7.8
**FM %**	36.9 ± 12.5	35.2 ± 11.4
**FFM %**	63.1 ± 12.5	62.7 ± 11.9
	**Comorbidities**	
	**Nutraceutical Group** **(n = 36)**	**Control Group** **(n = 32)**
**ULS grade**		
**Patients, n° (%)**		
Mild	4 (11.1)	6 (18.8)
Moderate	18 (50)	16 (50)
Severe	14 (38.9)	10 (31.2)
**Hepatic Steatosis Index**	49.3 ± 10.1	46.9 ± 7.6
**Diabetes n (%)**		
No prediabetes or diabetes	24 (66.7)	24 (75)
Prediabetes	12 (33.3)	8 (25)
Diabetes	0 (0)	0 (0)
	**Plasma Metabolic Parameteres**	
	**Nutraceutical Group** **(n = 36)**	**Control Group** **(n = 32)**
**Glucose, mg/dL**	105.6 ± 25.9	101.1 ± 22.5
**Total Cholesterol, mg/dL**	207.9 ± 49.7	213.1 ± 42.3
**HDL Cholesterol, mg/dL**	40.9 ± 13.8	59.4 ± 19.6 *
**LDL Cholesterol, mg/dL**	204.3 ± 56.9	180.5 ± 41.2 *
**LDL/HDL ratio**	5.6 ± 3.1	3.4 ± 1.4 *
**TG/HDL ratio**	4.3 ± 2.4	2.7 ± 1.8 *
**Triglyceride, mg/dL**	159.4 ± 65.9	133.8 ± 53.0
**AST**	38.5 ± 15.6	36.9 ± 13.0
**ALT**	53.8 ± 28.8	54.0 ± 21.1
	**Other Features**	
	**Nutraceutical Group** **(n =** **36)**	**Control Group** **(n = 32)**
**Current smoking n (%)**	26 (72)	22 (69)

**Abbreviations:** DS, standard deviation; T0, basal conditions; T1, after 3 months of treatment; BMI, body mass index (kg/m^2^); ULS, Ultrasound liver steatosis; AST, Aspartate-aminotransferase (UI/L); ALT, Alanin-aminotransferase (UI/L); TBW, total body water (%); FM, fat mass (%); FFM, fat-free mass (%).

**Table 3:** Anthropometric measurements as well as liver steatosis grade, biochemical parameters, body composition and hepatic steatosis index at baseline and after 3 months of treatment. Data are reported as mean ± DS. * *p* < 0.05 vs. T0; ° *p* < 0.05 vs. T1 Nutraceutical group.

	**Nutritional Assessment and Body Composition**			
	**Nutraceutical Group** **(n = 36)**		**Control Group** **(n = 32)**	
	**T0**	**T1**	**T0**	**T1**
**Body Weight, kg**	100.3 ± 19.2	92.8 ± 19.6 *	92.1 ± 17.8	84.7 ± 12.2 *
**BMI, kg/m^2^**	36.7 ± 8.6	34.0 ± 8.4 *	33.9 ± 6.6	31.8 ± 5.7 *
**Waist circumference, cm**	110.0 ± 15.4	103.7 ± 14.9 *	104.1 ± 11.3	97.5 ± 11.0 *
**Hip circumference, cm**	117.8 ± 13.9	114.0 ± 12.1 *	113.1 ± 13.7	110.3 ± 11.8 *
**TBW %**	46.9 ± 8.7	49.3 ± 9.2 *	48.5 ± 7.8	50.5 ± 7.3 *
**FM %**	36.9 ± 12.5	33.7 ± 13.6 *	35.2 ± 11.4	32.6 ± 10.7 *
**FFM %**	63.1 ± 12.5	66.0 ± 13.6 *	62.7 ± 11.9	67.3 ± 10.8 *
	**Comorbidities**			
	**Nutraceutical Group** **(n = 36)**		**Control Group** **(n = 32)**	
	**T0**	**T1**	**T0**	**T1**
**ULS grade**				
**Patients, n° (%)**				
Absent	0 (0)	6 (16.6)	0 (0)	0 (0)
Mild	4 (11.1)	20 (55.6)	6 (18.8)	14 (44)
Moderate	18 (50)	10 (27.8)	16 (50)	16 (50)
Severe	14 (38.9)	0 (0)	10 (31.3)	2 (6)
**Hepatic Steatosis Index**	49.3 ± 10.1	43.3 ± 9.0 *	46.9 ± 7.6	45.9 ± 6.9
	**Plasma Metabolic Parameteres**			
	**Nutraceutical Group** **(n = 36)**		**Control Group** **(n = 32)**	
	**T0**	**T1**	**T0**	**T1**
**Glucose, mg/dL**	105.6 ± 25.9	93.0 ± 15.4 *	101.1 ± 22.5	92.8 ± 13.8 *
**Total Cholesterol, mg/dL**	207.9 ± 49.7	161.4 ± 31.4 *	213.1 ± 42.3	187.7 ± 36.0 *°
**HDL Cholesterol, mg/dL**	40.9 ± 13.8	42.9 ± 13.9	59.4 ± 19.6	58.6 ± 11.6
**LDLCholesterol, mg/dL**	204.3 ± 56.9	143.0 ± 37.1 *	180.5 ± 41.2	150.2 ± 31.7 *°
**LDL/HDL ratio**	5.6 ± 3.1	3.6 ± 1.6 *	3.4 ± 1.4	2.6 ± 0.6 *
**TG/HDL ratio**	4.3 ± 2.4	3.1 ± 1.6 *	2.7 ± 1.8	1.8 ± 0.6 *
**Triglyceride, mg/dL**	159.4 ± 65.9	122.9 ± 43.4 *	133.8 ± 53.0	105.5 ± 35.4 *
**AST**	38.5 ± 15.6	25.0 ± 9.4 *	36.9 ± 13.0	24.6 ± 17.4 *
**ALT**	53.8 ± 28.8	29.4 ± 9.7 *	54.0 ± 21.1	38.3 ± 10.6 *

**Abbreviations:** DS, standard deviation; T0, basal conditions; T1, after 3 months of treatment; BMI, body mass index (kg/m^2^); liver steatosis grade: 0 = absent, 1 = mild, 2 = moderate, 3 = severe; ULS, Ultrasound liver steatosis; AST, Aspartate-aminotransferase (UI/L); ALT, Alanin-aminotransferase (UI/L); TBW, total body water (%); FM, fat mass (%); FFM, fat-free mass (%).

## Data Availability

The data are stored in a database at the Department of Clinical Medicine and Surgery, Nutrition Physiology Unit, University Federico II of Naples, Naples, 80131 Italy. It is available upon request to be made to Prof. Bruna Guida who a co-authors of the paper.
